# Classification of the Sidewalk Condition Using Self-Supervised Transfer Learning for Wheelchair Safety Driving

**DOI:** 10.3390/s22010380

**Published:** 2022-01-05

**Authors:** Ha-Yeong Yoon, Jung-Hwa Kim, Jin-Woo Jeong

**Affiliations:** 1 Department of Data Science, Seoul National University of Science and Technology, Seoul 01811, Korea; hi.yeong@seoultech.ac.kr; 2 Research Center for Data Science, Seoul National University of Science and Technology, Seoul 01811, Korea; junghwa.kim@seoultech.ac.kr

**Keywords:** deep neural networks, transfer learning, self-supervised learning, wheelchair safety

## Abstract

The demand for wheelchairs has increased recently as the population of the elderly and patients with disorders increases. However, society still pays less attention to infrastructure that can threaten the wheelchair user, such as sidewalks with cracks/potholes. Although various studies have been proposed to recognize such challenges, they mainly depend on RGB images or IMU sensors, which are sensitive to outdoor conditions such as low illumination, bad weather, and unavoidable vibrations, resulting in unsatisfactory and unstable performance. In this paper, we introduce a novel system based on various convolutional neural networks (CNNs) to automatically classify the condition of sidewalks using images captured with depth and infrared modalities. Moreover, we compare the performance of training CNNs from scratch and the transfer learning approach, where the weights learned from the natural image domain (e.g., ImageNet) are fine-tuned to the depth and infrared image domain. In particular, we propose applying the ResNet-152 model pre-trained with self-supervised learning during transfer learning to leverage better image representations. Performance evaluation on the classification of the sidewalk condition was conducted with 100% and 10% of training data. The experimental results validate the effectiveness and feasibility of the proposed approach and bring future research directions.

## 1. Introduction

With the growth in the population of the elderly and the incidence of disorders requiring mobility assistance, the demand for wheelchairs has recently increased. According to the recent report on the wheelchair market share and forecast [1], the wheelchair market was valued at USD 4 billion in 2021 and is expected to reach USD 6.5 billion by 2028, with a CAGR of 6.8%. However, a large number of wheelchair users are still challenged by insufficient urban infrastructure, such as the lack of wheelchair ramps and damaged sidewalk or roads, resulting in significant difficulties in their daily lives [2]. To address this issue, various studies and services have been presented. Studies from [3,4,5,6,7] attempted to improve and enhance the hardware utility and performance of a wheelchair. For example, Favey et al. and Arnay et al. [3,4] developed new sensors to increase the driving quality of electric wheelchairs, while studies from [5,6,7] focused on the development of motors and controllers to address various issues while driving through uphill, ramp, and stairs. In addition, there have been studies to facilitate wheelchair control by sensing surface electromyography (sEMG) signals from the human arm to detect gestures [8] or by using printed pressure sensor units to identify and inform irregular and improper posture to prevent sitting-related health issues [9]. Moreover, in [10], the muscular activity of the user was measured through electromyography (EMG) sensors, which were then processed and utilized to control both the wheelchair and robotic manipulator. Kim et al. [11] used electroencephalography (EEG) signals to establish a connection between brainwaves and three wheelchair commands: turn-left, turn-right, and move-forward. While previous work has improved the capabilities and functionalities of hardware and software technologies for a wheelchair, damaged urban infrastructure will still remain unmaintained or neglected without adequate public services. In this context, various applications and services were proposed by [12,13,14,15,16]. To detect and report urban anomaly events, some studies [12,13,14] utilized crowdsourcing mechanisms. Studies from [15,16] developed web/mobile-based applications to share issues regarding the maintenance of urban infrastructures. Despite the emergence of these services, people with disabilities still have to exert considerable effort if they wish to immediately report such issues to government offices by mobile or web applications while controlling or manually driving their wheelchairs.

With a recent growth of computer vision and machine learning technologies, there have been various attempts to automatically detect and report defects on roads and sidewalks. Previous approaches primarily captured RGB road images or sensor data (e.g., accelerometer and gyroscope) and exploited deep learning and machine learning algorithms for both detecting road cracks/potholes [17,18,19,20] and recognizing sidewalk anomalies [21,22]. These methods can automatically detect the defects on the road surface but still have the following limitations: (1) the captured RGB images are not helpful to classify the road condition under low-light conditions (e.g., nighttime) and (2) sensors can produce noisy data or restrict the user’s natural movements, adversely affecting the overall performance. Therefore, studies on advanced techniques are still required to achieve more robust performance as well as improved usability.

In this paper, we propose a novel system to automatically classify sidewalk conditions using depth and infrared imaging modalities to handle the aforementioned issues. The proposed system monitors the sidewalk surface by downward recording using a single camera attached to the wheelchair and uses an advanced deep learning-based technique to achieve a robust performance. Specifically, the captured images are used for training a ResNet-152 [23] architecture using a self-supervised transfer learning approach. To exploit the advanced image representation learned from self-supervised learning, pre-trained weights on the ImageNet [24] dataset are used through the SimCLRv2 framework [25], which is one of the state-of-the-art self-supervised learning (SSL) approaches. For performance evaluation, we compare the classification accuracy of the proposed approach with those of supervised learning and supervised transfer learning methods, and analyze how the image modality (i.e., depth, infrared, and depth+infrared) affects the overall performance.

The main contributions of this paper are twofold:

(1) We investigated the feasibility of adopting a self-supervised representation learning and transfer learning approach for classifying the condition of the sidewalk. In particular, it was demonstrated that image representations learned from the general image domain (e.g., ImageNet) can be applied to the domain of sidewalk images.

(2) We evaluated the performance of our approach based on the single-modal (i.e., depth or infrared) data as well as multi-modal (i.e., depth+infrared) data. For the multi-modal approach, we exploited both early fusion (i.e., combining raw images) and late fusion (i.e., combining intermediate CNN features) methods. Through the experimental result, we discussed how the choice of image modality affects the performance of the proposed approach.

The rest of this paper is organized as follows: Section 2 describes the data collection procedure and Section 3 provides details of the proposed approach. In Section 4, an analysis of the experimental result is presented. Finally, the conclusions, limitations, and further research directions are discussed in Section 5.

## 2. Data Collection

**Figure 1:**
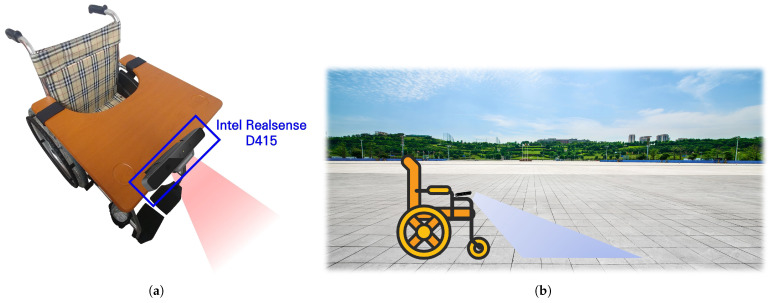
Wheelchair setup. (**a**) Hardware setup, (**b**) Recording configuration.

To establish a dataset for our study, we set up the hardware configuration of a wheelchair, as shown in Figure 1a. A single Intel RealSense D415 camera that supports multi-modal recording with depth and infrared modalities was used to capture sidewalk images. As shown in Figure 1a, the camera was installed on the desk of the wheelchair for downward recording. Figure 1b illustrates our recording configuration while driving the wheelchair. The images of the surface of sidewalks in front of the wheelchair (30–50 cm away) were recorded at 3–5 frames per second.

**Figure 2:**
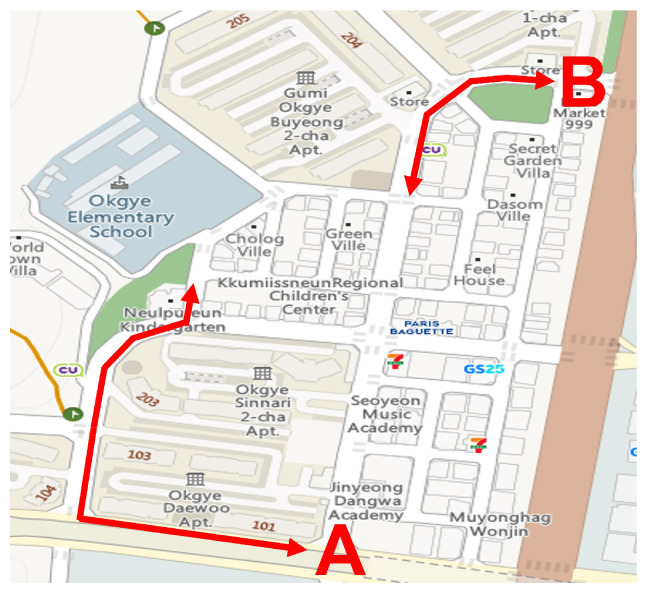
Recording route.

For data collection, six university students (3 male and 3 female, 22–24 years old) were recruited to drive a wheelchair. Figure 2 shows the predetermined route for the data collection. The route consisted of two sub-routes, namely (A) and (B), as shown in Figure 2, comprised of straight and curved pavements. The data collection procedure consisted of 2 sessions corresponding to each sub-route. Specifically, the participants moved through the sidewalk between the endpoints of each sub-route and took a 10 min break between the sessions. During data collection, the participants drove the wheelchair at a normal speed (i.e., approximately 0.77 m/s). While driving the wheelchair along the route, a pair of depth and infrared images were captured simultaneously over a period of 30–40 min for each subject.

**Figure 3:**
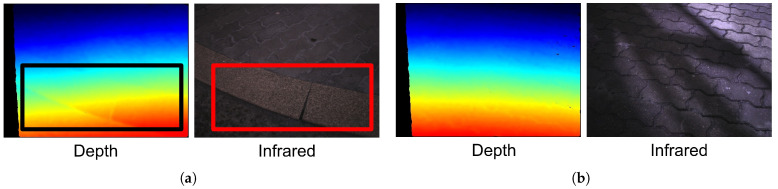
Example of sidewalk images. (**a**) Damaged sidewalk, (**b**) Normal sidewalk.

As a result, we collected 1500 images of damaged sidewalks and another 1500 images of normal sidewalks for each modality (i.e., depth and infrared). Examples of the captured images can be found in Figure 3. Unlike RGB images, which may not be useful under low-light conditions (e.g., at dawn or night) [26,27] or bad weather (cloudy or rainy conditions) [28,29], images captured with the modalities used in this study are relatively less affected by outdoor conditions [30]. Therefore, it is expected that the use of depth and/or infrared modality images will facilitate the classification of sidewalk conditions in the wild. While collecting the images, we could observe some physical shocks and vibrations caused by wheelchair users’ rough driving skills and/or bad sidewalk conditions applied to the wheelchair body, which may degrade the quality of the images. In this work, however, only the raw images without any image preprocessing steps applied were used for training and testing the models to figure out the effectiveness of the deep learning-based approaches. Nevertheless, the raw images in Figure 3 still clearly show the difference between the images of damaged and normal sidewalks. In contrast to the normal sidewalk (see Figure 3b), the curbs and cracks (black and red boxes in Figure 3a) resulted in irregular patterns on both the depth and infrared images. In using the images with a single modality (i.e., depth or infrared) or image pairs with both modalities, the CNN models were trained with various learning strategies to classify the condition of the sidewalk as either normal or damaged. In the next section, we describe the details of how we trained a CNN model to classify the condition of sidewalks using the collected data.

## 3. Classification of Sidewalk Condition

**Figure 4:**
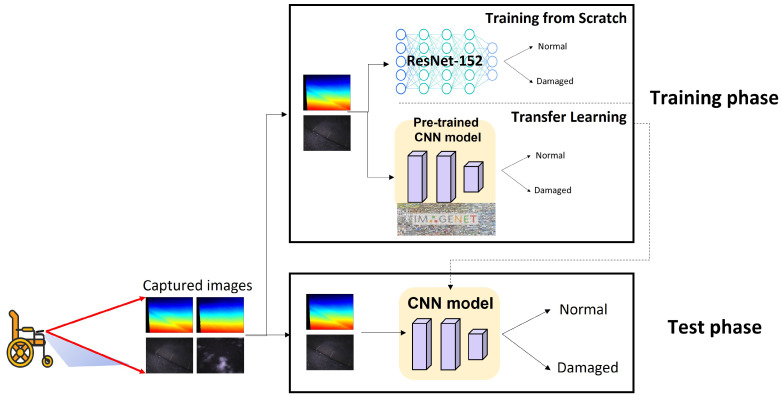
System overview.

Figure 4 depicts an overview of the proposed system which consists of training and testing phases. As mentioned in Section 2, a set of images of sidewalks were captured with depth and infrared modalities, and were then used for both training and testing CNNs. As shown in the testing phase of Figure 4, the problem to be addressed in this paper is a binary classification task in which a label of each sidewalk image with various modalities (i.e., depth, infrared, and depth+infrared) is classified as either normal or damaged. In the course of CNN training, we built three different CNN training pipelines using the following strategies: (1) supervised learning from scratch and (2) transfer learning with pre-trained models. In particular, for a transfer learning approach, we utilized (1) the models pre-trained on the ImageNet dataset with supervised labels and (2) the models pre-trained on the ImageNet dataset without labels (i.e., models trained with self-supervised learning). For each learning approach, we also exploited a multi-modal approach in which a set of image pairs of depth and infrared modalities were used for training and testing. For all the pipelines, we exploited ResNet-152 architecture [23] as our base network architecture. Finally, the trained models from each different strategy were used in the testing phase for the performance evaluation. The next subsections describe the details of each learning approach.

### 3.1. Supervised Learning from Scratch

**Figure 5:**
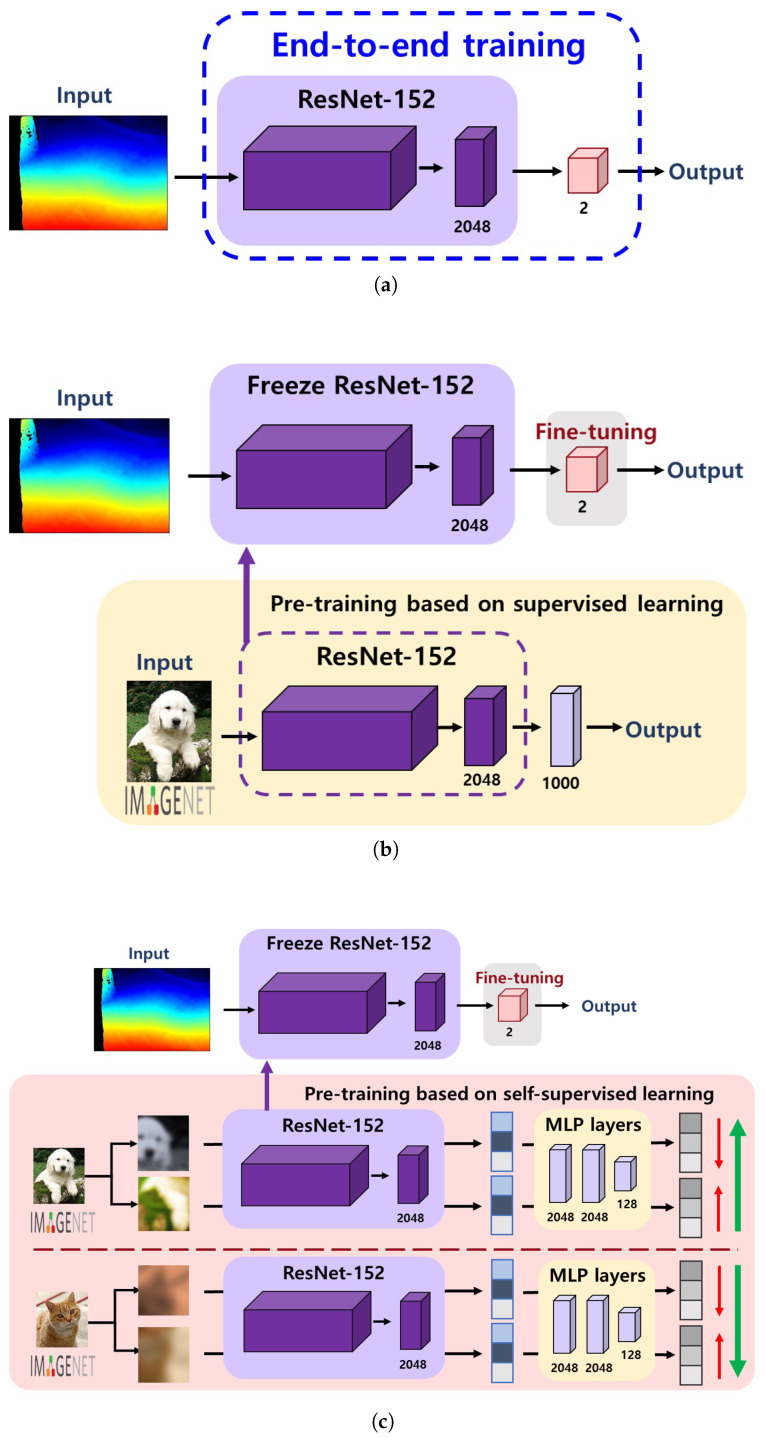
Training strategies. (**a**) Supervised learning from scratch, (**b**) Transfer learning with supervised pre-trained models, (**c**) Transfer learning with self-supervised pre-trained models.

Supervised learning from scratch is a standard method for training a base model (e.g., CNN in our case) with randomly initialized weights. For this strategy, a set of image–label pairs for the target domain should be prepared. Figure 5a illustrates the procedure for supervised learning from scratch applied in this study. As depicted in the figure, we used the ResNet-152 as a base network architecture, which is a model that won first place at the ILSVRC 2015 classification task and reported a 3.57% error on the ImageNet dataset. As shown in Table 1, the ResNet-152 architecture consists of five convolution blocks with 152 layers. The convolution blocks were designed with 1 × 1 and 3 × 3 convolution kernels, except for the Conv1 block. During the training process, images from the target domain (i.e., depth or infrared sidewalk images) and their corresponding labels were used as input data. Therefore, the network directly learned the image features from the dataset and classified each image as either normal or damaged.

### 3.2. Transfer Learning with Supervised Pre-Trained Models

Transfer learning is a well-known approach to utilize the weights of an existing model pre-trained on a large-scale dataset (e.g., ImageNet dataset) rather than to update the weights from scratch to solve the same or similar task. With a fine-tuning task where the pre-trained weights are updated to fit the target domain, the model can be more quickly converged even with higher accuracy [31]. For this strategy, we used the ResNet-152 network pre-trained on the ImageNet database, followed by a single dense layer to be updated for our domain. Figure 5b shows how a transfer learning process with supervised pre-trained models works. In contrast to the supervised learning from scratch, where the initial random weights are used, the network first adopts the weights learned from a set of image–label pairs in the ImageNet dataset and then fine-tunes the final layer to classify the condition of sidewalk images. Since the pre-trained model functions as a feature extractor in this protocol, all the layers in the pre-trained model are frozen, while only the final layer is kept trainable.

### 3.3. Transfer Learning with Self-Supervised Pre-Trained Models

In contrast to the above approach, the SSL approach does not require class labels while learning image representation during a pre-training task. Instead, the SSL solves various pretext tasks without labels [32,33,34,35], such as an instance discrimination task where the features of the same instance are pulled away from those of all other instances [36]. This is also based on the idea that under a certain type of image augmentation, the learned representations should be invariant; therefore, the network can implicitly learn the underlying structure/representation of the data. For the SSL-based transfer learning, we used the ResNet-152 model pre-trained on the ImageNet database with the SimCLRv2 framework [25], which is one of the state-of-the-art SSL methods for image classification.

**Figure 6:**
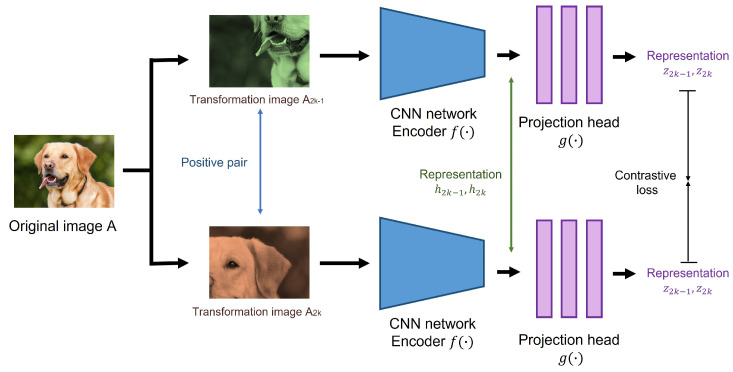
Pre-training process of SimCLRv2.

SimCLRv2 adopts a contrastive learning approach for learning underlying image representations without class labels. Figure 6 briefly shows a pre-training process of SimCLRv2. First, the model uses a total of *N* mini-batch examples to perform random crop, color distortion, and Gaussian blur on each image $x_{i}$ twice. The transformed images ($x_{2k-1},x_{2k}$) from the same image are called positive pairs. The image representations ($h_{2k-1},h_{2k}$) of the images are then extracted by ResNet-152 encoder f(·). Representations are transformed to features ($z_{2k-1},z_{2k}$) by passing through the projection head g(·) MLP networks. Finally, the model attempts to find a set of representations for the positive pair by using the following contrastive loss:

(1) $$l_{i,j}=-log\frac{exp(sim\left(z_{i},z_{j}\right)/\tau )}{\sum_{k=1}^{2N}{𝟙}_{\left[k\neq i\right]}exp\left(z_{i},z_{k}\right)/\tau }$$

where *i* and *j* indicate a positive pair of the same image, ${𝟙}_{\left[k\neq i\right]}$ is a indicator function used as 1 when [k≠i], τ denotes a temperature parameter, and sim(·,·) is a cosine similarity between two vectors. Compared to the first version of SimCLR [33], there have been several design changes applied to fully leverage the power of general pre-training. For example, SimCLRv2 increased the capacity of the projection head g(·) by making it a deeper non-linear network; replaced the base network (ResNet-50) with a deeper but less wide model; and replaced ResNet-152(3×) with 3× wider channels, selective kernels, and a channel-wise attention mechanism that improved the parameter efficiency of the network.

Figure 5c illustrates the workflow of transfer learning with self-supervised pre-trained models. The network first adopts the weights learned from the SimCLRv2 self-supervised learning pipeline, which attempts to learn the underlying image representations of the ImageNet dataset, and then fine-tunes the subsequent layers to classify the condition of sidewalk images. Similar to the transfer learning approach with supervised pre-trained models, the pre-trained ResNet-152 model is used as a feature extractor only; therefore, all the layers in the pre-trained model are frozen, while only the final layer is kept trainable.

### 3.4. Multi-Modal Learning

In this study, we designed (1) a single-modal approach and (2) two types of multi-modal fusion approaches for each training strategy as follows.

**Figure 7:**
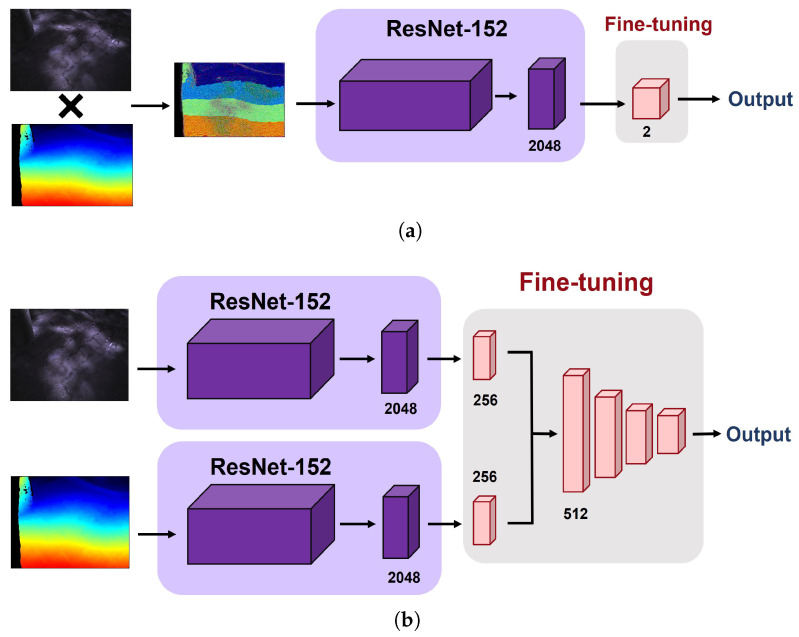
Workflow of early fusion and late fusion approaches. (**a**) Early fusion, (**b**) Late fusion.

- Single-modal approach: Similar to the general CNN architecture for image classification, the single-modal approach only takes a set of single-modal images (i.e., depth or infrared) as input for the network.
- Multi-modal approach: Compared to the single-modal approach, a set of multi-modal images (i.e., pairs of depth and infrared images) are fed into the network in this case. To this end, we applied early fusion (i.e., combining raw images) and late fusion (i.e., combining intermediate CNN features) methods. For early fusion, we conducted element-wise multiplication between the infrared and depth images in the same pair before feeding the images into the network. As depicted in Figure 7a, therefore, only a single pipeline is required for this type of multi-modal learning. For late fusion, we first extracted 256-dimension features from each modality and then concatenated them into a single 512-dimension feature vector. This final feature vector is then passed to the subsequent dense layers for classification of the condition of sidewalks. Figure 7b depicts the procedure of late-fusion between the depth and infrared images.

## 4. Experiments

### 4.1. Experimental Setup

In this paper, the experiments were conducted on a high-end server equipped with a single Geforce RTX 2080Ti GPU, 32GB RAM, and an Intel i7-10700K CPU. We used the Tensorflow framework to implement the proposed system.

For the experiment with single-modal images, we randomly selected 2000 images as the training set and another 1000 images as the testing set. Similarly, for the experiments with multi-modal images, 2000 depth and infrared image pairs were used as a training set and another 1000 pairs were used as a testing set. The original images with a resolution of 640 × 480 were resized to 224 × 224 and then used for the training and testing of deep neural networks for classifying sidewalk conditions.

For the supervised learning from scratch (called Supervised hereafter) and transfer learning with supervised pre-trained models (called Transfer_supervised_ hereafter), the SGD optimizer with a learning rate of 0.0001 was used. With transfer learning with self-supervised pre-trained models (called Transfer_SSL_ hereafter) using late fusion, the SGD optimizer with a learning rate of 0.0005 was used. In the case of Transfer_SSL_ with single-modal data and early fusion approaches, the lars optimizer [37] with a learning rate of 0.0001 was used. All the models were trained for 300 epochs with a batch size of 10, except Supervised with a late fusion approach (5).

### 4.2. Evaluation

In the experiment, we evaluated the performance of the proposed method trained with different learning strategies. In particular, to validate the robustness and effectiveness of self-supervised learning, we divided our dataset into the full dataset containing 100% of the training samples and a subset containing only 10% of the training samples and compared the performance of each method on both datasets. All reported values were averaged from 10 repetitive experiments.

First, we discuss the classification accuracies of each model on the full dataset. Table 2 summarizes the validation accuracy of each model trained with 100% of the training data (i.e., 2000 images for single-modal and 2000 image pairs for multi-modal setups). The numbers in the table represent the mean accuracies and standard deviations. From Table 2, we can observe the following results:

(1) The supervised learning from scratch approach showed the worst performance among the classification models. Specifically, it achieved a validation accuracy of 65.81% for the depth and 57.45% for the infrared modality. The use of multi-modal data was not helpful in increasing the performance of the Supervised approach, yielding 61.71% and 53.56% for the early and late fusion, respectively. It should be noted that this approach failed to achieve a high accuracy although it only utilized a set of images with labels from the target domain (i.e., road surface images). This can be due to the insufficient amount of data available for training a network which has a number of trainable parameters. This is also in line with the common observation that the supervised learning of CNNs from scratch requires a large amount of data from the target domain to have a successful performance [38].

(2) All the classification models based on transfer learning outperformed the supervised learning from scratch model. Specially, Transfer_supervised_ achieved a performance gain of 2.57%, 14.92%, 4.77%, and 16.32% in the depth-based, infrared-based, early fusion, and late fusion approaches, respectively. Additionally, the Transfer_SSL_ approaches showed a higher performance improvement of 4.77%, 14.07%, 6.42%, and 21.3% in the depth-based, infrared-based, early fusion, and late fusion approaches, respectively. These results validate the feasibility of utilizing the transfer learning approach based on the ImageNet database for our domain. It is also worth noting that the weights from the model pre-trained on the image dataset consisting of RGB images of general objects were effective for the depth/infrared images of the surface of sidewalks.

(3) The Transfer_SSL_ methods yielded performances comparable to or even better than Transfer_supervised_, even though they were based on the image representations learned from various pretext tasks without any image/class labels. Specifically, the depth-modality and early fusion approaches produced 1.65–2.2% better accuracies than Transfer_supervised_. Furthermore, the multi-modal approach with late fusion achieved the highest accuracy of 74.86%, outperforming all the other approaches. This implies that transfer learning using image representations/features learned from self-supervision tasks on a dataset containing objects and modalities that are significantly different from our target domain also works and can produce promising results. Since collecting training data for self-supervision tasks that do not require labels is relatively easy, we can also expect further performance improvement from enhanced image representations at a low cost.

(4) We found that a multi-modal fusion approach does not always work. The early fusion approach was not helpful in improving the performance of the training methods used in this study. No performance improvement was observed from the Supervised and Transfer_supervised_ approaches even though the late fusion was applied. Specifically, there was an average performance degradation of 4.0% for Supervised, 2.2% for Transfer_supervised_, and 2.9% for Transfer_SSL_ with early fusion. Only Transfer_SSL_ when adopting a late fusion approach achieved a higher performance over single-modal approaches. It was also found that transfer learning-based approaches, which exploit the weights of the models pre-trained for learning image representations, resulted in a better performance with a late fusion approach (i.e., feature-level fusion) than with the early fusion approach. In sum, with 100% of the training data, we could observe the best classification accuracy using Transfer_SSL_ based on multi-modal data with a late fusion approach. Finally, the confusion matrices of all the networks trained with 100% of the training data can be found in Figure A1, Appendix A.

Second, to validate the effectiveness of image representations learned from self-supervised learning, we also conducted a performance evaluation using only 10% of the training data (i.e., 200 images for single-modal images and 200 image pairs for multi-modal images). Table 3 summarizes the validation accuracy of each model trained with 10% of the training data. As expected, we could see that the performance of all the models drastically decreased as the amount of training data reduced. In particular, the Supervised approach reached an almost chance level. Transfer_supervised_ achieved an accuracy of 58–62%, which is approximately 8% less than the model trained with 100% data on average. The performance of the single-modal-based Transfer_SSL_ approach also decreased to 63.32% with a 7.73% drop on average, while they were still better than the Supervised (52.85% on average) and Transfer_supervised_ (61.37% on average) approaches. Most notably, Transfer_SSL_ with early fusion did not significantly suffer from a reduced amount of training data, yielding the highest accuracy of 64.45%. In contrast, we could observe a large performance drop of Transfer_SSL_ with the late fusion approach, from 74.86% (with 100% data) to 62.55% (with 10% data), which is, however, still better than the other approaches. For more details, the confusion matrices of all the networks trained with 10% of the training data are presented in Figure A2, Appendix A.

**Figure 8:**
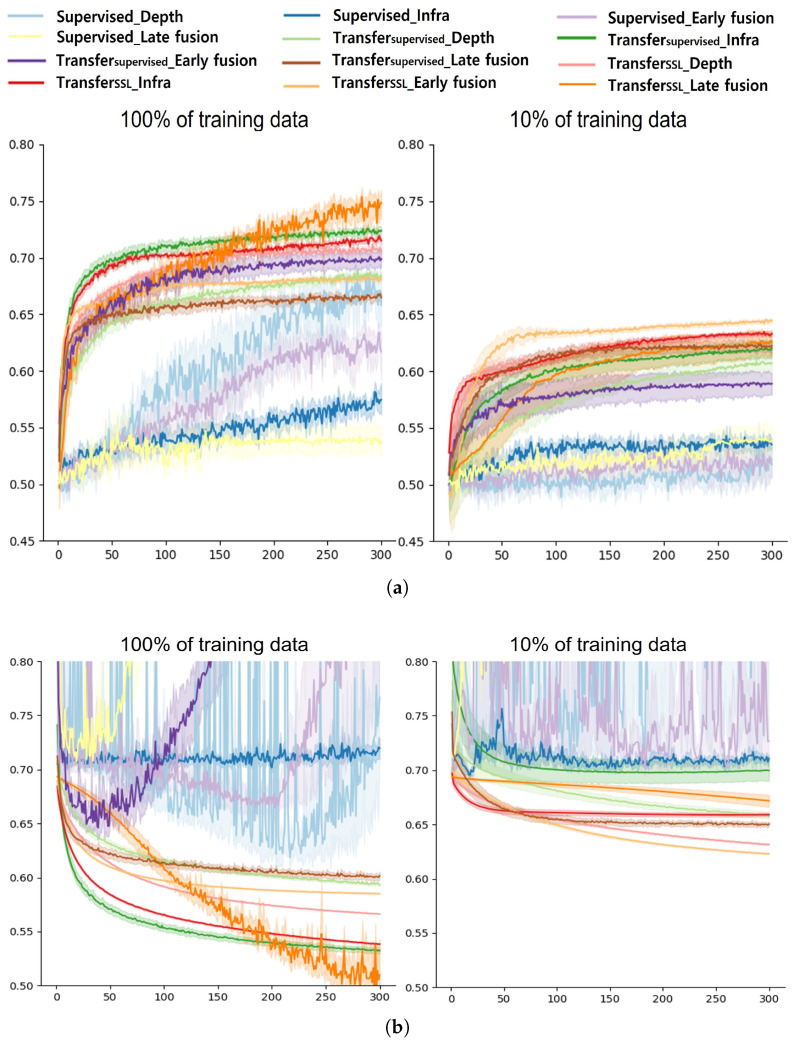
Accuracy and loss of each method per epoch. (**a**) Comparison of validation accuracy, (**b**) Comparison of validation loss.

**Figure A1:**
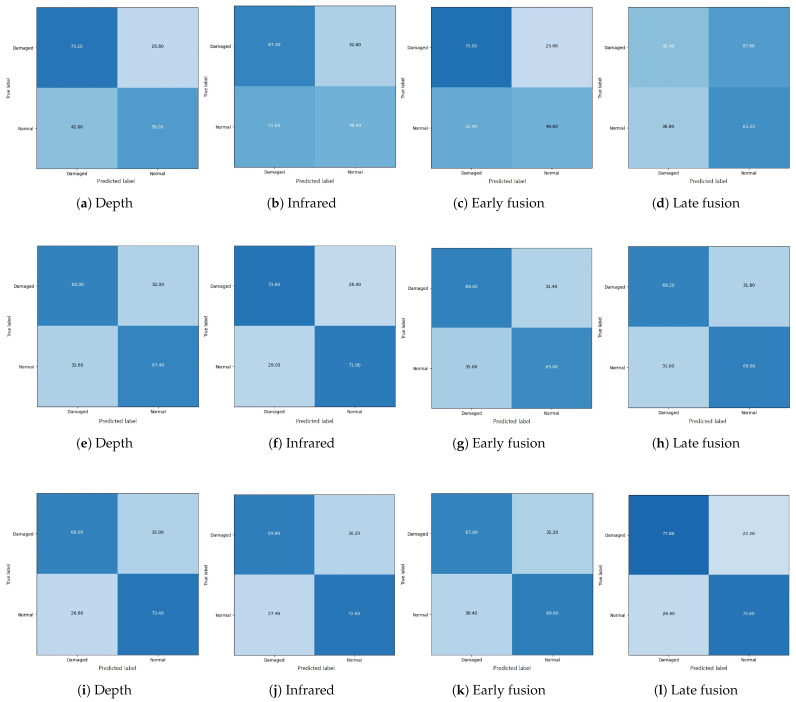
Confusion matrix with 100% of training data: the first, second, and third row indicate the confusion matrices from the Supervised, transfer_supervised_, and transfer_SSL_ approaches, respectively.

**Figure A2:**
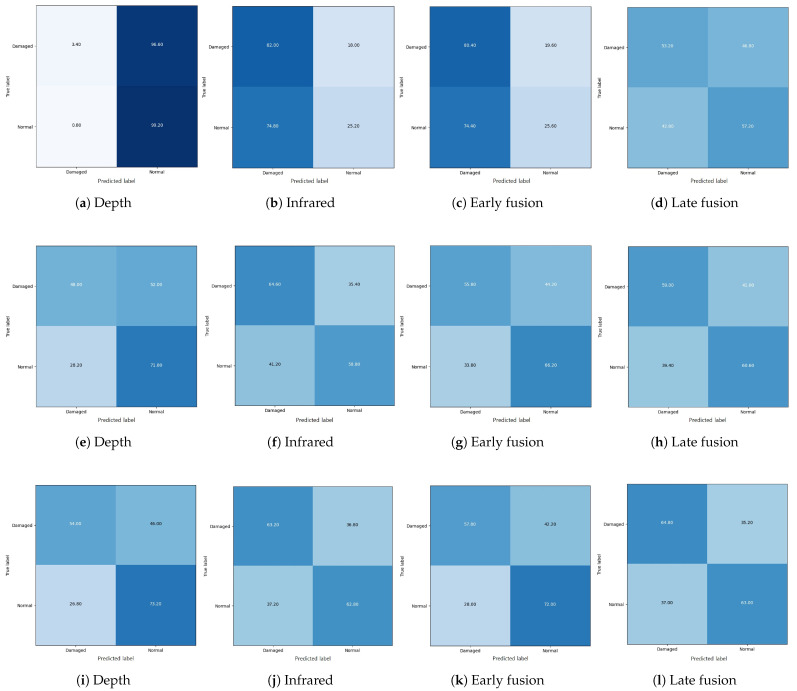
Confusion matrix with 10% of training data: the first, second, and third row indicate the confusion matrices from the Supervised, transfer_supervised_, and transfer_SSL_ approaches, respectively.

Table 4 summarizes the performance differences according to the amount of training data. Generally, transfer learning-based approaches showed less performance drops compared with Supervised approaches. It seems that Supervised with an infrared modality and a late fusion approach was less affected by the reduced training data; however, its performance was close to the chance level accuracy for both 100% and 10% data, which is not meaningful. In contrast, the Transfer_SSL_ approaches tended to show competitive accuracy with less performance drops, resulting in a more robust performance. However, as noted above, the late fusion approach of Transfer_SSL_ failed to preserve a high classification accuracy when the amount of training data was limited. This result is also related to the number of trainable parameters for each method, as summarized in Table 5. The Supervised approach attempts to learn the features from scratch with a large number of trainable parameters (58 M); therefore, a large amount of training samples are essential for a successful training. As a result, the Supervised methods presented a large performance drop as well as the lowest accuracy (chance level) in our experiment. Transfer_SSL_ with a late fusion approach requires more trainable parameters as well as a more complicated architecture than other approaches, resulting in difficulties in training a model with a limited amount of data. Finally, Figure 8a,b show the validation accuracy and loss of each model per epoch for both 100% and 10% training data setups. As shown in the figures, the Supervised approaches failed to produce a stable performance while transfer learning-based approaches worked better for both cases.

Finally, Table 6 summarizes the inference time required for each method. It was shown that the most complex architectures (i.e., networks trained with multi-modal late fusion) consumed more time to make prediction results. Based on the result, we believe that our frameworks are efficient enough to be used in real-time scenarios (i.e., with at least 25–34 FPS) and can be more optimized by further enhancement.

## 5. Discussion and Conclusions

In this work, we proposed a novel sidewalk condition recognition system for wheelchair users using depth and infrared images, as well as various deep learning techniques. Our experimental findings showed that self-supervised learning with multi-modal data achieved the best performance regardless of the amount of training data and validated the feasibility of the proposed method. In addition to the quantitative evaluation, we briefly compared our work with the previous studies in terms of qualitative aspects and discussed how our approach works differently. Table 7 summarizes the differences among the studies working on the automatic classification/detection of defects on roads and sidewalks. As shown in Table 7, most studies attempted to utilize RGB images and acceleration data for recognizing road conditions. However, studies from [17,18,19,20] mainly focused on detecting the damages of a motorcar road and required a smartphone to be installed on the dashboard of a vehicle, thus the method is not suitable for wheelchair users. In contrast, Watanabe et al. and Iwasawa et al. [21,22] tried to recognize the status of the sidewalk by using acceleration data. However, this kind of data is not only largely sensitive to the outdoor conditions and inherent vibrations/noises of a wheelchair but also not feasible to provide users with intuitive information about the defects observed. Moreover, collecting and labeling a large amount of wheelchair vibration data for training machine learning or deep learning models is another hurdle that must be overcome. To address the limitations of previous studies, we utilized multi-modal images captured by a single camera that can be installed to the body of a wheelchair and applied a transfer learning approach with pre-trained models which learned visual features from unlabeled data using a self-supervised learning strategy. We showed that fine-tuning the models pre-trained on the general image domain, using the self-supervised learning strategy, to the heterogeneous image domain (i.e., depth and infrared sidewalk images) works successfully. In addition, we found that the image features learned in a self-supervised way better convey underlying image representations, thus the proposed method could achieve more stable and robust performances even if the number of training samples was reduced when compared to the models of traditional learning strategies. We believe the proposed work shows a promising approach for a domain where the amount of heterogeneous multi-modality samples is limited, in particular.

However, there still exists room for improvement in terms of classification accuracy and functionality. First, in this study, we attached a depth camera to the wheelchair desk for recording forward scenes, but this configuration cannot be applied to the wheelchair without a desk option. However, there are still several alternatives that can be considered. In the case of manual wheelchairs, a camera can be installed at the front or side frame of the armrest (or body) of the wheelchair. In contrast, electric wheelchairs are generally equipped with a controller pad to drive the wheelchair, thus the front edge of the controller can be considered one of the best places to install the camera. In particular, multiple cameras can be used together for recording and recognizing sidewalk conditions in the case a power supply issue is not critical. Second, our approach utilized only a single camera for the classification of sidewalk conditions; however, the number and position of the installed cameras can be changed according to the type of wheelchair. Recently, various approaches based on multi-view images (i.e., images from multiple cameras) have been presented to improve the performance of pose estimation and object recognition [39,40,41]. Inspired by this, we expect that the proposed method can be extended to exploit multi-view images for better performance. To this end, we also plan to apply model compression or pruning algorithms to optimize the network architectures, minimizing the computing resources (e.g., power consumption, memory usage, etc.) required for the real-time processing on edge devices. Third, our current work cannot visualize/display damaged regions/routes on the map because it focuses on the classification of sidewalk conditions. Therefore, we will utilize GPS sensor data in the future to visualize the route where the wheelchair users move away as well as a set of regions where severe damages were observed.

In sum, there still exist various challenging issues to be addressed; therefore, we will extend our study by establishing a large dataset from more users and by training CNNs with various setups. We also believe that our approach can be adapted to personal mobility vehicles, such as electric kickboards and bicycles, thereby improving driver safety in the future.

## Figures and Tables

**Table 1:** Architecture of ResNet-152.

Layer Name	Output Size	152-Layer
Conv1	112 × 112	7 × 7, 64, stride 2
Conv2	56 × 56	3 × 3 max pool, stride 2
		$\left[\begin{array}{c} 1\times 1,64\\ 3\times 3,64\\ 1\times 1,256 \end{array}\right]\times 3$
Conv3	28 × 28	$\left[\begin{array}{c} 1\times 1,128\\ 3\times 3,128\\ 1\times 1,512 \end{array}\right]\times 8$
Conv4	14 × 14	$\left[\begin{array}{c} 1\times 1,256\\ 3\times 3,256\\ 1\times 1,1024 \end{array}\right]\times 36$
Conv5	7 × 7	$\left[\begin{array}{c} 1\times 1,512\\ 3\times 3,512\\ 1\times 1,2048 \end{array}\right]\times 3$
	1 × 1	Average pool, 1000-d fc, softmax

**Table 2:** Validation accuracy on 100% of training data (unit: %).

Training Method	Depth	Infrared	Early Fusion	Late Fusion
Supervised	65.81 ± 0.0316	57.45 ± 0.0238	61.71 ± 0.0356	53.56 ± 0.0178
Transfer_supervised_	68.38 ± 0.0052	72.37 ± 0.0047	66.48 ± 0.0063	69.88 ±0.0111
Transfer_SSL_	70.58 ± 0.0038	71.52 ± 0.0052	68.13 ± 0.004	74.86 ± 0.0206

**Table 3:** Validation accuracy on 10% of training data (unit: %).

Training Method	Depth	Infrared	Early Fusion	Late Fusion
Supervised	52.18 ± 0.0280	53.52 ± 0.0073	51.76 ± 0.0209	53.86 ± 0.0208
Transfer_supervised_	60.72 ± 0.0101	62.02 ± 0.0113	62.24 ± 0.0076	58.88 ± 0.0161
Transfer_SSL_	63.35 ± 0.0045	63.29 ± 0.0032	64.45 ± 0.0025	62.55 ± 0.0173

**Table 4:** Performance loss according to the amount of training data (unit: %).

Training Method	Depth	Infrared	Early Fusion	Late Fusion
Supervised	13.63	3.93	9.95	−0.3
Transfer_supervised_	7.66	10.35	4.24	11
Transfer_SSL_	7.23	8.23	3.68	12.31

**Table 5:** Number of trainable parameters.

Method	Single-Modal	Early Fusion	Late Fusion
Supervised	58,223,618	58,223,618	117,564,194
Transfer_supervised_	4098	4098	1,125,154
Transfer_SSL_	4098	4098	1,125,154

**Table 6:** Inference time (unit: FPS).

Method	Depth and Infrared	Early Fusion	Late Fusion
Supervised	204	142	34
Transfer_supervised_	207	142	26
Transfer_SSL_	188	208	25

**Table 7:** Comparison with previous studies.

Ref.	Target	Measuring Devices	Measured Data	Number of Modalities	Classifier	Learning Method
[17]	Road condition	Smartphone	Acceleration and gyroscope	2	ML	Supervised learning
[18]	Road condition	Smartphone	RGB images	1	DL	Supervised learning
[19]	Road condition	Smartphone	Acceleration	1	ML	Supervised learning
[20]	Road condition	Smartphone and RGB camera	RGB images	1	DL	Supervised learning
[21]	Sidewalk condition	Three-axis accelerometer	Acceleration	1	DL	Weakly supervised learning
[22]	Sidewalk condition	Three-axis accelerometer	Acceleration	1	ML	Supervised learning
Ours	Sidewalk condition	Depth camera	Depth and infrared images	2	DL	Self-supervised learning

## Data Availability

Not applicable.
